# Comprehensive Analysis of Potential Biomarkers of Acute Lymphoblastic Leukemia in Children by Using a Competing Endogenous RNA Network

**DOI:** 10.1155/2022/4563523

**Published:** 2022-04-06

**Authors:** Xiao-Ying Lin, Ka-Yuk Yuen, Hai-Lei Chen, Miao-Na Shen, Yan Huang, Qian-Wen Huang, Yong Liu, Lu-Hong Xu

**Affiliations:** ^1^Hematologic Laboratory of Pediatrics, Sun Yat-sen Memorial Hospital, Sun Yat-sen University, Guangzhou 510120, China; ^2^Guangdong Provincial Key Laboratory of Malignant Tumor Epigenetics and Gene Regulation, Sun Yat-sen Memorial Hospital, Sun Yat-sen University, Guangzhou 510120, China; ^3^Department of Pediatrics, Sun Yat-sen Memorial Hospital, Sun Yat-sen University, Guangzhou 510120, China

## Abstract

Acute lymphoblastic leukemia (ALL) is the most serious hematological carcinoma in adolescents. The significance of long noncoding RNAs (lncRNAs) and their regulative role in the proliferation and differentiation of myeloid cells in cancer has been recently reported. Nevertheless, key RNAs and the regulatory mechanism of competitive endogenous RNA (ceRNA) network affected by pediatric ALL are not fully illustrated. In this study, phase 2 and 3 pediatric ALL RNA profiles were extracted from the TARGET database and used to identify lncRNAs, microRNAs, and messenger RNAs in high-risk ALL and reconstruct the sponge ceRNA regulatory network. Results indicated that 44 lncRNAs, 25 miRNAs, and 115 mRNA were up/downregulated. Functional analysis with differentially expressed RNAs (DERNAs) showed enriched significant signaling pathways, including PI3K-Akt and p53 signaling cascades and other pathways associated with the tumor. Seventeen differential hub RNAs, including LINC00909, BZRAP1-AS1, C17orf76-AS1, HCG11, MIAT, SNHG5, SNHG15, and TP73-AS1, were identified. The Cox model of correlation indicated that 14 of these RNAs were associated with the progression of pediatric ALL. These findings would help clarify the regulatory role of several lncRNAs as well as provide insights into the leukemogenesis of pediatric ALL to further explore novel prognostic markers/therapeutic targets for ALL.

## 1. Introduction

Acute lymphoblastic leukemia (ALL) is a major type of serious carcinoma in children and adolescents [1]. It remains the most frequent cause of death from cancer in individuals aged 20 years or younger [2]. ALL is characterized by abnormal proliferation and differentiation of lymphoid progenitor cells. Although chemotherapy, remission-induction therapy, and hematopoietic cell transplantation of ALL can increase the 5-year survival rate to about 90%, the prognosis after relapse is undesirable [3]. Therefore, novel prognostic markers/therapeutic targets for ALL must be identified.

Long noncoding RNAs (lncRNAs) are a functional RNA molecule with length of 200 nucleotides. Increasing lines of evidence have supported the idea that lncRNAs are involved in lymphoid and hematopoietic differentiation and proliferation. lncRNA CASC15 can mitigate the expression of the transcriptional activator SOX4 in RUNX1-translocated leukemia possibly by modulating the activity of transcription factors, such as YY1 [4]. The increased expression of lncRNA HOTAIR as well as EZH2, LSD1, DNMT3A, and DNMT3B is closely associated with poor prognosis in patients with acute leukemia [5]. The transcription factor TCF7L2 increases the expression of downstream lncRNA HOXA-AS2, which modulates resistance to prednisone and leads to enhanced proliferation and inhibited apoptosis in ALL cells [6].

The hypothesis of competing endogenous RNAs (ceRNAs) was proposed to explain the regulatory mechanism of interactions between RNAs in ALL. Guo et al. [7] analyze lncRNAs in human chronic granulocytic leukemia by the lncRNA cDNA microarray. They found that lncRNA-BGL3 is a key regulator of BCR-ABL-mediated cell transformation. Further experiments demonstrated that lncRNA-BGL3 acts as ceRNA to bind and interact with these miRNAs to regulate phosphatase expression. miRNAs, such as miRNA-126 [8], miRNA-155 [9], and miR-141-3p [10] can regulate specific functions in ALL. ceRNAs work as a miRNA sponge to control miRNA. However, the interplay between lncRNAs and miRNAs in terms of the ceRNA regulatory network in ALL requires further clarification.

Understanding lncRNA-miRNA-mRNA causal relationships in hematological tumors would help elucidate the underlying regulatory mechanisms in cancers, such as ALL and AML. lncRNA SBF2-AS1 facilitates the cell proliferation of AML via sponged miR-188-5p in the abrogation of repression of ZNF91 [11]. lncRNAs NEAT1 and SMC1A comodulate cell proliferation and apoptosis in AML by cooperatively suppressing the expression of miR-23a-3p [12]. UCA1 works as a miR-16 sponge to regulate MDR1 [13]. In diffuse large B-cell lymphoma (DLBCL), the evidence shows that cell proliferation is impeded by direct binding of lncRNA SMAD5-AS1 to miR-135b-5p, resulting in the increased expression of APC and the decreased activation of the Wnt/Beta-catenin pathway [14]. lncRNA CCAT1 induces cell arrest and promotes cell apoptosis to modulate HOXA1 expression by working as a ceRNA against miR-181a-5p in multiple myeloma [15]. However, the ceRNA regulatory network in response to pediatric ALL has not been comprehensively analyzed.

In this study, we focused on identifying hub lncRNAs in pediatric ALL by using the expression profiles from the TARGET database. ceRNAs, especially lncRNAs between high- and low-risk groups of pediatric ALL, were prioritized. lncmiRSRN was used to construct the differential lncRNA-miRNA-mRNA ceRNA regulatory network and identify differential hub lncRNAs between the high- and low-risk groups of pediatric ALL. Results indicated a differential hub ceRNA network that consists of 44 lncRNAs, 25 miRNAs, and 115 mRNAs. The lncRNAs in the ceRNA network were significantly associated with biological processes and cancer signaling pathways in pediatric ALL. Furthermore, lncRNAs (LINC00909, BZRAP1-AS1, C17orf76-AS1, HCG11, LINC01004, MIAT, RP4-717I23.3, RP11-77H9.2, RP11-94L15.2, RP11-311C24.1, RP11-473M20.16, SNHG5, SNHG15, and TP73-AS1) were identified in pediatric ALL. Survival analysis indicated that these hub lncRNAs were significantly correlated with the prognosis of children with ALL. This work provides insights to identify potential diagnostic/therapeutic targets of ALL.

## 2. Materials and Methods

### 2.1. Data Resource and Preprocessing

The level 3 expression profiles (raw counts) of RNAs and miRNAs of pediatric ALL samples were acquired from the Therapeutically Applicable Research to Generate Effective Treatment (TARGET) database (https://ocg.cancer.gov/programs/target/data-matrix). lncRNAs and mRNAs were reannotated with an in-house python script. The script was designated for the ensemble of ID extraction from the gene matrix profile and subsequently annotated to a gene symbol with reference to ENCODE human ver.36. ceRNAs without the gene symbol were removed. The average expression of replicated lncRNAs and mRNAs was obtained to determine a unique value for calculation.

Clinical data, including endpoint vital status, overall survival (OS) duration and time, and laboratory test results, were obtained (up to November 28, 2021). The exclusion criteria were as follows: (i) phase 2/3 samples with a cell of origin from a B-cell precursor, (ii) samples without RNA and miRNA sequencing data, and (iii) samples without clinical test results including gene fusion status, white blood cell (WBC) count, and OS period. A criterion of WBC ≥ 50 was set for risk stratification. The expression profiles of 261 pediatric ALL samples (169 low-risk samples and 92 high-risk samples) were included. R packages survminer and survplot were used to analyze high-risk childhood ALL. lncRNAs, miRNAs, and mRNAs were identified with annotation to ENCODE ver.36. The corresponding clinical information of the 92 high-risk samples and 169 low-risk samples is available publicly from the TARGET program Lymphoblastic Leukemia project (https://ocg.cancer.gov/programs/target/projects/acute-lymphoblastic-leukemia). Thus, a waiver of ethics approval from the local ethics committee was implicated.

### 2.2. Analysis of Differentially Expressed Genes (DEG)

The “Deseq” package in R 4.0.0 was used to identify differentially expressed RNAs in childhood ALL. *P* values were corrected with the false discovery rate (FDR) to avoid multitesting error. The significance of DERNAs was set with criteria of FDR < 0.01 and absolute value of log2fold change > 1 [16, 17]. All three types of DERNAs (lncRNAs, miRNAs, and mRNAs) were visualized in a volcano plot by using the ggplot package in R.

### 2.3. Construction of the ceRNA Sponge Regulatory Network

A bioinformatics analysis pipeline of lncmiRSRN was used to identify an lncRNA-related sponge regulatory network in childhood ALL. The pipeline utilizes functions to construct a sponge regulatory network with lncRNA and mRNA expression data by integrating putative miRNA-target and miRNA-lncRNA interactions.

lncmiRSRN was featured by multiple built-in R functions including the QueryTarget binding function to predict candidate miRNA-target interaction by integrating putative miRNA-mRNA and miRNA-lncRNA interactions from the expression data. Candidate miRNA-mRNA interactions were extracted using several experimentally validated databases: miRTarbase v7.0 and TarBase 7.0 for miRNA-target interaction; NPInter v3.0 and LncBase v2.0 for miRNA-lncRNA interactions; and LncRmR for lncRNA-mRNA pairs from the putative miRNA sponge interactions. The significance of common miRNAs shared by each lncRNA-mRNA pair was determined by calculating the hypergeometric distribution in the validated lncRNA-mRNA interactions (*M* > 2 and <0.01). The *P* value for positively correlated lncRNA-mRNA pairs was calculated and adjusted by Benjamin–Hochberg (BH) method.

lncmiRSRN was used to identify lncRNA-related miRNA sponge interactions across high-risk and low-risk childhood ALL groups by prioritizing the intersection of shared lncRNA-mRNA pairs. The pairs denote the significance of the positive correlation in the sponge regulatory network. lncmiRSRN then estimated the causal effects of sponge lncRNAs on mRNAs. For the inferred positively correlated lncRNA-mRNA pairs, the causal effect between the sponge lncRNA and mRNA was measured with a scoring function by using the Benjamin–Hochberg- (BH-) adjusted *P* value. The lncRNA-miRNA sponge regulatory network was tested with Fisher's asymptotic *P* value function from the R package WGCNA to evaluate the strength of the lncRNA-mRNA interaction. By assembling the causal relationship, we established causal lncRNA-related miRNA sponge regulatory networks between the high- and low-risk samples of childhood ALL.

Considering that the regulatory ceRNA network may be perturbed by different pathology causes, we identified hub lncRNAs by evaluating the topological properties in the causal sponge lncRNA-mRNA interactions for childhood ALL. For each node in lncmiRSRN, its degree is defined as the number of edges connected with it. The node degree often falls into a power law distribution in a scale-free network. This principle is one of the fundamental metrics in interpreting actual biological networks. Hub genes with higher degrees tend to be critical; as such, lncmiRSRN selects the top 20% of lncRNAs with the highest degree as hub lncRNAs. Different lncRNA-mRNA regulatory relationships between high-risk and low-risk childhood ALL were compared. A score of dissimilarity was calculated and presented in a graph to determine the association of hub lncRNAs and sponge lncRNA-mRNA regulatory relationships. The gene modules in different lncmiRSRN networks in both groups were addressed using the Markov clustering algorithm (MCL) in the R package ProNet.

### 2.4. Functional and Pathway Enrichment Analyses by GO and KEGG

Enrichment analysis of the coding genes of different hub lncRNAs to the biological process (BP) category of Gene Ontology (GO) and Kyoto Encyclopedia of Genes and Genomes (KEGG) pathways was performed using the clusterProfiler package to identify biological activities and signaling pathways associated with mRNAs in the ceRNA sponge regulatory network. The significant terms enriched in BP, CC (cellular component), and MF (molecular function) were identified and visualized with the R package Chord and ggplot2. *P* value < 0.05 was the significance threshold for the enriched terms.

### 2.5. Analyses of Risk Association of Hub lncRNAs, miRNAs, and mRNAs and Prognosis and Survival of Children with ALL

The 261 phase 2/3 childhood ALL samples were independent of one another, and related clinical data, such as miRNA and mRNA expression, prognosis, and OS time, were available in TARGET. The Predict.coxph function in the survival package was used to calculate risk scores. Survival curves were generated between hub lncRNA expression and OS time of pediatric patients with ALL by using the Kaplan–Meier method. *P* < 0.05 was considered the threshold for significance in the analyses.

## 3. Results

### 3.1. Demographic Data of Pediatric Patients with ALL

**Figure 1:**
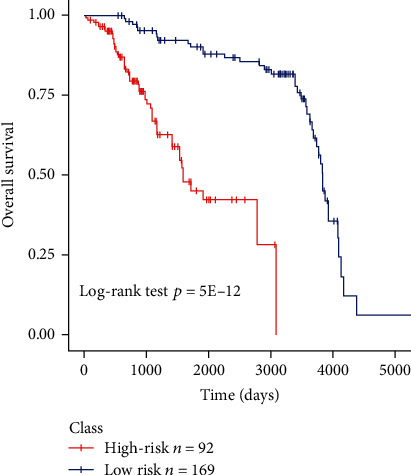
OS rate in the high-risk group with pediatric ALL.

The clinical data and demographics of pediatric ALL samples are presented in Table 1. A total of 261 patients had B-precursor ALL, including 169 low-risk samples and 92 high-risk samples. The median age of the samples was 6.6 years, and no significant difference in gender (male to female ratio: 1.04) was found. According to St. Jude's guideline for risk stratification, patients were assigned into high- and low-risk groups with the criterion of WBC count ≥ 50. The OS rate in the high-risk group was significantly lower than that in the low-risk group (*P* value = 5*E* − 12, Figure 1).

### 3.2. Identification of DElncRNA, DEmiRNA, and DEmRNA from Pediatric ALL Samples

**Figure 2:**
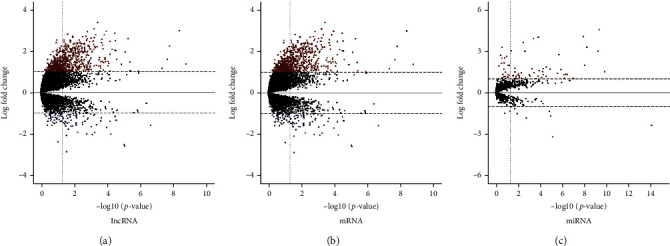
Volcano plots showing the number, significance, and FDR of DERNAs between the high- and low-risk groups of pediatric ALL. A threshold of ∣log2foldchange | >2 and FDR < 0.05 was set for significant DEGs. The red dots indicate upregulated DERNAs, and blue dots indicate downregulated lncRNAs (a), mRNAs (b), and miRNAs (c). The *x*-axis represents the adjusted FDR, and the *y*-axis indicates the value of log2foldchange.

Differentially expressed ceRNAs with ∣log2FC | >1 and adjusted *P* value < 0.01 were identified. The ceRNAs included 1267 mRNAs, 565 lncRNAs, and 41 miRNAs between the high- and low-risk groups. The upregulated RNAs included 1091 mRNAs, 393 lncRNAs, and 25 miRNAs, while the remaining RNAs were downregulated. The distribution of ceRNAs and their expression were visualized as scattered dots on the volcano plot (Figure 2).

### 3.3. Construction of the ceRNA Sponge Regulatory Network and Identification of Hub lncRNA by Using lncmiRSRN

A sponge lncRNA-mRNA regulatory network was constructed by lncmiRSRN using the high- and low-risk childhood ALL samples. A total of 565 lncRNAs and 2,518 mRNAs were used. The number of lncRNA-mRNA sponge regulatory interactions differed between the high-risk and low-risk childhood ALL samples. The 16,050 candidate lncRNA-miRNA pairs and 6,401 candidate miRNA-mRNA pairs from the experimentally validated sources were incorporated to build the sponge lncRNA-mRNA regulatory network.

Causal effects were scored in the high- and low-risk ALL groups. In the high-risk group, the causal scores of 231 lncRNAs on 430 mRNAs ranged from –11.086 to 12.947, and 75,454 positive effects were identified, accounting for 47.9%. In the low-risk group, the causal scores of 65 mRNAs and 57 lncRNAs ranged from –1.69 to 2.301 (*P* value of 0 and 1), with 55.4% positive causal effects. The strength of the causal effects was evaluated with Fisher's asymptotic test, with adjusted *P* value cutoff threshold of 0.05. The higher positive causal effects than the negative ones may suggest that mRNAs are always upregulated by sponge lncRNAs in childhood ALL.

To determine the significance of the lncRNA-mRNA regulatory relationship in high-risk ALL, we evaluated different and conserved lncRNA-mRNA regulatory relationships between the two groups. The majority of sponge lncRNA-mRNA regulatory relationships (~62%) and hub lncRNAs (17, ~50%) were shared by the high- and low-risk childhood ALL. A considerable proportion of common miRNA sponge regulatory relationships was identical in the biological processes of high- and low-risk childhood ALL.

**Figure 3:**
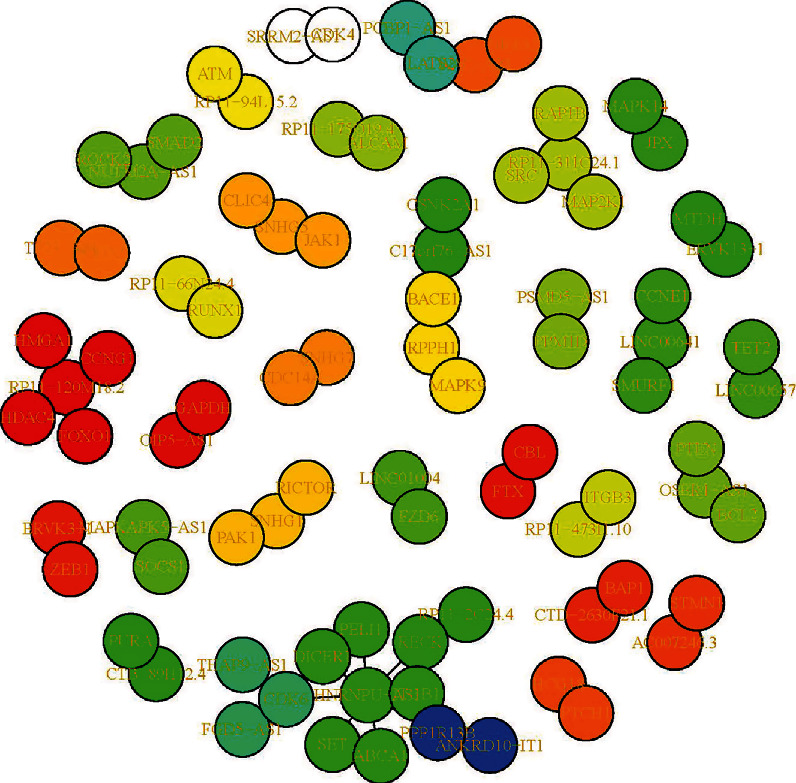
lncRNA-mediated differential ceRNA regulatory network in high-risk pediatric ALL. The green dots represent lncRNAs, the yellow dots represent miRNAs, and the red dots represent mRNAs.

The different and conserved lncRNA-mRNA regulatory networks were visualized with the R package i-graph based on the degree of connection in the ceRNA regulatory network (Figure 3). The color of nodes represents the three types of RNAs (lncRNAs, miRNAs, and mRNAs), and the closeness between the nodes was evaluated as the interactions. RNAs with a higher degree of connection were prioritized as hub RNAs, which established the causal lncRNA-mRNA regulatory pairs, and hub lncRNAs in high-risk childhood ALL were revealed.

The likeness between each lncRNA-mRNA pair was scored to evaluate the similarity of the lncRNA-mRNA regulatory network and hub lncRNAs between high- and low-risk ALL. The similarity score between lncRNA-mRNA pairs was high in the high- and low-risk ALL groups (Sim = 0.622). The similarity of hub lncRNAs was also assessed. In contrast to the network similarity, the dissimilarity between high- and low-risk ALL was relatively moderate, with a value of 0.333. Seventeen differentially expressed hub lncRNAs were identified to find the pivotal lncRNAs behind high-risk ALL. Hence, certain lncRNAs may selectively play a role in poor prognosis in childhood ALL.

To evaluate the pivotal RNAs in different sponge lncRNA-mRNA regulatory relationships, we investigated the differential biological functions of lncRNA-mRNA pairs between high- and low-risk childhood ALL. Based on the experimentally validated gene-disease associations, 17 lncRNAs and 131 mRNAs in differential lncmiRSRNs were closely associated with high-risk childhood ALL. Specifically, hub lncRNAs NUTM2A-AS1, LINC00641, OSER1-AS1, and HNRNPU-AS1 were found to be involved in high-risk childhood ALL. The hazard ratio between the two groups is 0.333, and the log-rank *P* value is close to 0. Thus, differentially expressed lncRNAs and mRNAs can act as prognostic factors because of their significant association with metastasis risk in pediatric ALL.

### 3.4. Functional Enrichment Analysis of Differential lncRNAs and mRNAs in Childhood ALL

**Figure 4:**
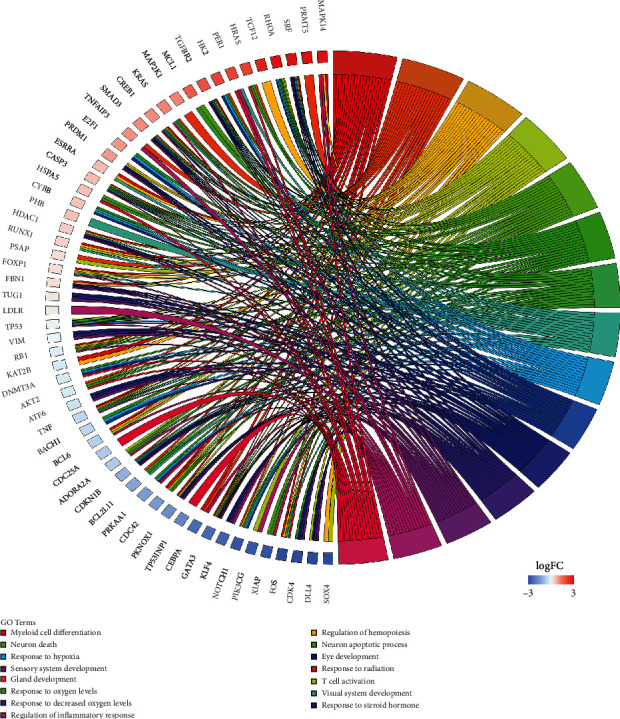
Enriched BP terms of GO from lncRNAs involved in the ceRNA network of pediatric ALL.

In terms of the biological process and pathways of dysregulated mRNAs in lncmiRSRNs, 131 mRNAs were subjected to GO functional analysis and KEGG enrichment analysis. The terms enriched in the top 15 BP categories included “myeloid cell differentiation” (adjusted *P* value = 2.25*E* − 03), “regulation of hemopoiesis” (adjusted *P* value = 1.22*E* − 02), “response to hypoxia” (adjusted *P* value = 1.36*E* − 08), and “regulation of inflammatory response” (adjusted *P* value = 8.01*E* − 08), which are closely associated with the progression of childhood ALL (Figure 4). The most enriched terms in the MF category of GO were “DNA-binding transcription factor binding” (adjusted *P* value = 7.18*E* − 05), “RNA polymerase ii-specific DNA-binding transcription factor binding” (adjusted *P* value = 1.17*E* − 03), “chemokine receptor binding” (adjusted *P* value = 2.84*E* − 03), and “DNA-binding transcription repressor activity” (adjusted *P* value = 1.67*E* − 02).

**Figure 5:**
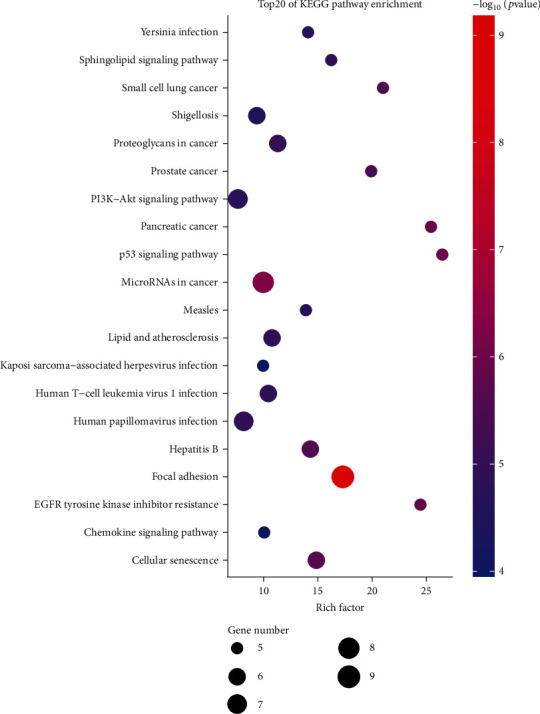
Top 20 enriched KEGG pathways of RNAs involved in the ceRNA regulatory network in pediatric ALL.

**Figure 6:**
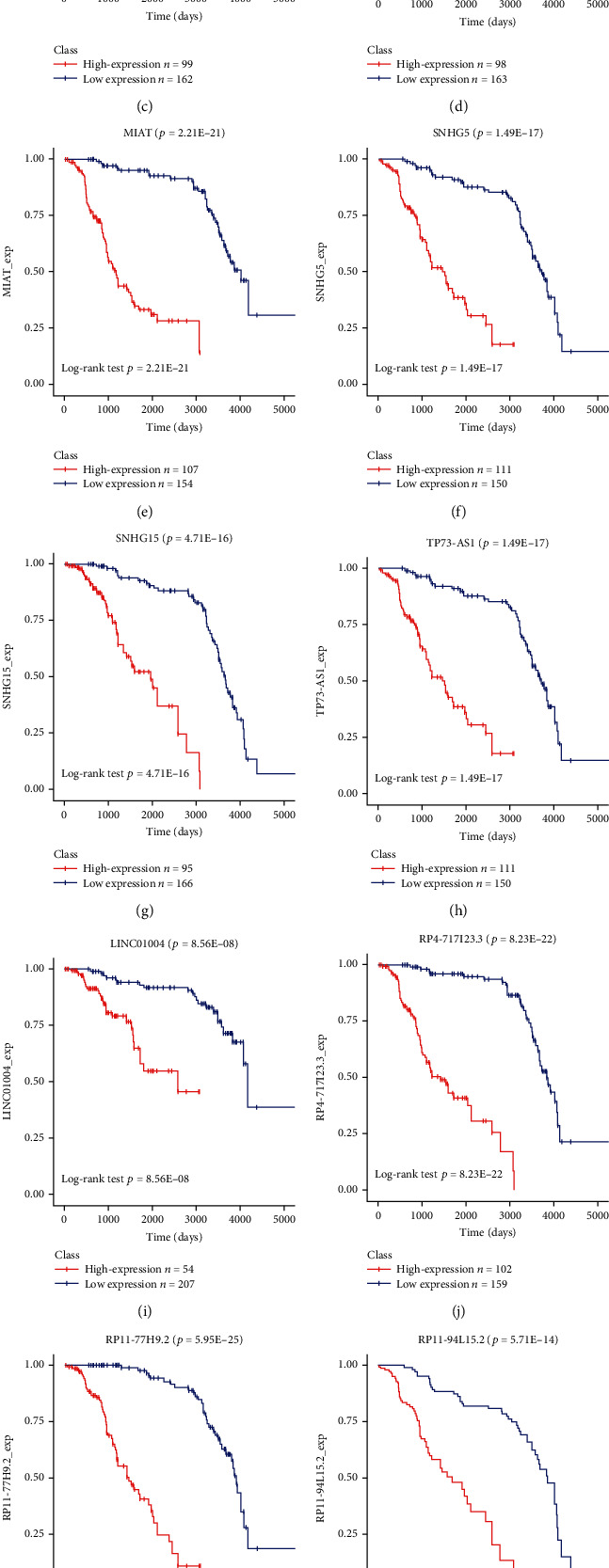
Survival curves of 14 differential hub lncRNAs that were significantly correlated with the OS of pediatric patients with ALL in the ceRNA network.

The analysis of the enrichment of the 131 dysregulated mRNAs in the KEGG database indicated the presence of potential altered biological functions between high- and low-risk ALL groups. The top 20 significantly enriched signaling pathways are presented in Table 2 and Figure 5. The most enriched pathways included “microRNAs in cancer,” “PI3K-Akt signaling pathway,” “p53 signaling pathway,” “focal adhesion,” and “chemokine signaling pathway,” which are involved in the progression and metastasis of ALL. Cancer-related pathways, such as “small-cell lung cancer,” “proteoglycans in cancer,” “prostate cancer,” and “human T-cell leukemia virus 1 infection,” were also enriched, suggesting the robust involvement of biological functions identified from dysregulated mRNAs in childhood ALL. The result of KEGG pathway enrichment is presented as a bubble chart in Figure 6.

### 3.5. Assessment of the Relationship of Prognosis and OS to Differential Hub lncRNAs and mRNAs in Pediatric ALL

OS was analyzed with the KM method to determine the association between differential hub lncRNAs and the prognosis of childhood ALL (Figure 6). The threshold of *P* < 0.05 was set as the criterion of significance. The survival analysis reveals that 14 lncRNAs (82.3%) of the 17 differential hub lncRNAs significantly determined the metastasis risk of pediatric ALL (hazard ratio > 2, log-rank *P* value < 0.01). These lncRNAs included the following: LINC00909 (*P* = 8.66*E* − 19), BZRAP1-AS1 (*P* = 2.26*E* − 13), C17orf76-AS1 (*P* = 1.96*E* − 13), HCG11 (*P* = 1.08*E* − 14), LINC01004 (*P* = 8.56*E* − 08), MIAT (*P* = 2.21*E* − 21), RP4-717I23.3 (*P* = 8.23*E* − 22), RP11-77H9.2 (*P* = 5.95*E* − 25), RP11-94L15.2 (*P* = 5.71*E* − 14), RP11-311C24.1 (*P* = 5.28*E* − 13), RP11-473M20.16 (*P* = 4.15*E* − 18), SNHG5 (*P* = 1.49*E* − 17), SNHG15 (*P* = 4.71*E* − 16), and TP73-AS1 (*P* = 1.49*E* − 17).

Most of the differential hub lncRNAs and their role in the related regulatory sponge relations seem to be cancer specific. A query to the lnc2Cancer database showed that eight of the 14 differential hub lncRNAs (LINC00909, BZRAP1-AS1, C17orf76-AS1, HCG11, MIAT, SNHG5, SNHG15, and TP73-AS1) were associated with various types of carcinomas in previous studies. Hence, differential hub lncRNAs may be proactively involved in the biological processes of pediatric ALL by regulating a similar lncRNA-miRNA-mRNA axis targeting other cancers.

The association between six lncRNAs (LINC01004, RP4-717I23.3, RP11-77H9.2, RP11-94L15.2, RP11-311C24.1, and RP11-473M20.16) and the prognosis of pediatric ALL was reported for the first time. The potential role of these lncRNAs in the sponge ceRNA regulatory network of ALL should be further verified.

**Figure 7:**
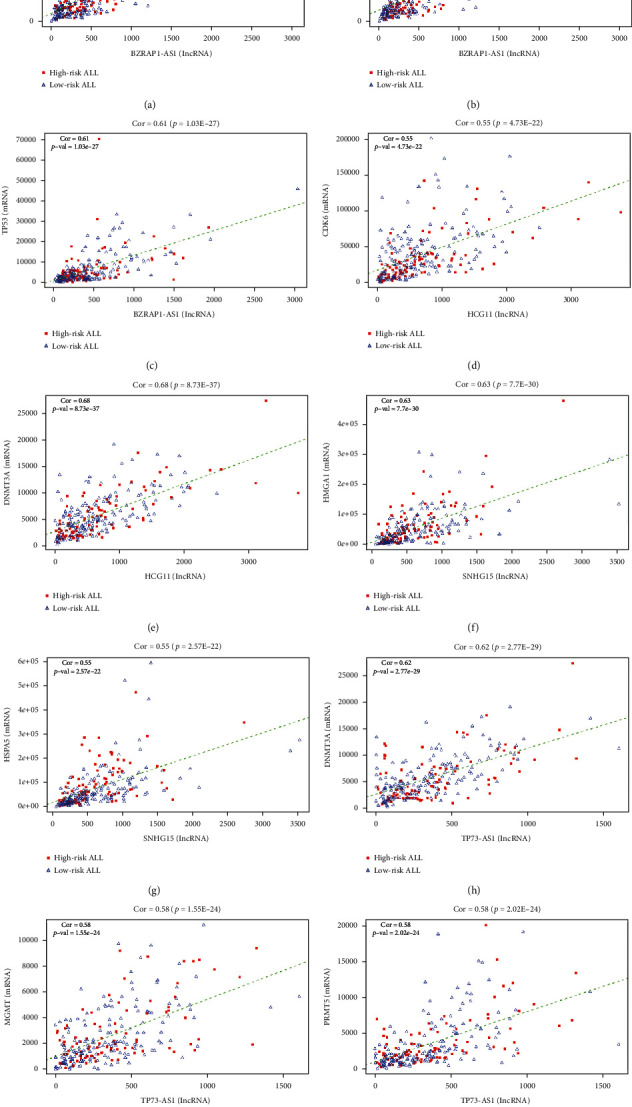
Pearson correlation analysis between the expression levels of differential hub lncRNAs and literature-validated mRNAs in the ceRNA network (Cor: correlation coefficient).

In ceRNA theory, lncRNAs may regulate mRNAs released by miRNAs. In this process, lncRNAs work as a miRNA sponge to coregulate mRNAs. A positive correlation possibly exists between mRNAs and lncRNAs modulated by identical miRNAs. This presumption was further tested in our sponge regulatory network. The expression of miRNAs, mRNAs, and 10 hub lncRNAs regulated by the released miRNAs was highly correlated, including BZRAP1-AS1, SNHG15, TP73-AS1, and 10 associated mRNAs (Figure 7). The Pearson correlation coefficient for the lncRNA-mRNA associations was more than 0.55, with *P* value less than 0.001. Our findings indicate that differential hub lncRNAs may serve as potential drivers that affect the prognosis of pediatric ALL.

## 4. Discussion

This study analyzed the RNA-seq data of pediatric ALL from the largest childhood cancer database TARGET. A differential ceRNA sponge regulatory network between high- and low-risk pediatric ALL was established, and differential hub lncRNAs were found. Among all lncRNAs in the differential ceRNA network, pairs of 231 lncRNAs on 430 mRNAs were found as differential. Further analysis confirmed that 14 of the 17 differential hub lncRNAs were closely associated with the OS of patients with ALL. The GO functional and KEGG pathway enrichment analyses for RNAs in the differential ceRNA network were performed. RNAs were mainly enriched in myeloid cell differentiation, regulation of hemopoiesis, DNA-binding transcription factor binding, RNA polymerase ii-specific DNA-binding transcription factor binding, and DNA-binding transcription repressor activity, similar to the report of Wang et al. [18]. The enrichment to the KEGG pathways indicated the involvement of several cancerous pathways, including microRNAs in cancer, PI3K-Akt signaling pathway, and p53 signaling pathway. Targeting the frequently altered p53 signaling pathway in ALL, especially the main regulators of p53, MDM2, and CDKN2A, is a novel therapeutic strategy in anticancer drug discovery [19]. The novel selective MDM2 inhibitor Nutlins (in phase III clinical trials) showed promising pharmaceutical properties in restoring the p53 pathway. In particular, nonfunctional missense mutations were frequently found in pediatric ALL in the DNA-binding domain of the tumor suppressor gene TP53 [20]. This finding suggests that the combination of the regimen of DNA damaging and p53 reactivation agents could be an effective antileukemia strategy. Evidence indicated that molecules in the PI3K-Akt signaling pathway mitigated in the proliferation and metastasis of ALL [21–23]. Promising drugs targeting the PI3K/Akt/mTOR signaling pathway, such as IGF1R inhibitors (NT157, OSI-906), might be another effective strategy for patients with B-precursor ALL [24]. Additionally, cancer-related pathways, such as small-cell lung cancer, proteoglycans in cancer, prostate cancer, and human T-cell leukemia virus 1 infection, were enriched. The GO terms associated with cancer immunology included regulation of inflammatory response and chemokine receptor binding. The KEGG pathway terms of focal adhesion and chemokine signaling pathway were also enriched. The CC chemokine receptor type 5 (CCR5) inhibitor maraviroc was proven to suppress growth and induce apoptosis in ALL at the cell level [25]. These results confirm the association between lncRNAs in the ceRNA network and biological processes in pediatric ALL.

Eight of the 14 differential hub lncRNAs have been identified to be associated with the progression of various tumors; most of these lncRNAs participate as sponges in mediating the miRNA-mRNA axis paradigm and affect potent therapeutic targets in pediatric ALL. SNHG15 could regulate cisplatin resistance in breast cancer by sponging miR-381 expression [26]. TP73-AS1 mediates the proliferation of breast cancer cells by inhibiting miR-200a to regulate the expression of TFAM [27]. In hematological cancers, such as AML, the overexpressed MIAT in AML functions as a sponge to inhibit miRNA-495 (miR-495) and abrogate the suppression of carcinogenesis [28]. The upregulated SNHG5-miR-489-3p axis impedes SOX4 expression, thereby suppressing cell proliferation and inducing apoptosis in AML [29]. HCG11 suppresses the growth of glioma by interaction with miR-4425 to express MTA3 [30]. The overexpressed LINC00909 promotes tumor progression in human glioma by regulating the miR-194/MUC1-C axis [31]. Hence, the identified differential hub lncRNAs may serve as crucial regulators of the pathogenesis of high-risk ALL.

This study has several limitations. Phase 2 and 3 clinical data initially included 261 pediatric ALL samples only, of which their miRNA and mRNA expression profiles were evaluated. The coverage of samples was not large, and only 14 positive findings of differential hub lncRNAs with pediatric ALL were obtained. We cannot eliminate the possibility that certain lncRNAs that may have a causal relationship to pediatric ALL are not included. Bioactivity and function tests of LINC01004 and five newly identified lncRNAs and assessment of their regulatory mechanism were not performed and should be further investigated. The RNA-seq profiles of ALL in our data contained tissues with the cell origin of B-cell precursor and had no other ALL subtypes, such as T-cell precursor. Nevertheless, we evaluated many literature-validated RNAs. Overall, lncRNAs may act as sponges in the ceRNA regulatory network of pediatric ALL and may lead to tumorigenesis.

In conclusion, we constructed a ceRNA network associated with childhood ALL and reported 17 hub lncRNAs as potential diagnostic biomarkers of high-risk childhood ALL. We also obtained 14 potent prognostic biomarkers, including six that were newly identified, for ALL with high OS. We demonstrated the contribution of lncRNA-mRNA interactions and the key roles of lncRNAs in the progression of pediatric ALL. Further investigation into the molecular mechanism of the discovered sponged lncRNAs will facilitate the clinical diagnosis and treatment of pediatric ALL.

## Figures and Tables

**Table 1:** Clinical characteristics of 261 patients with pediatric ALL.

	Low risk (*N* = 169)	High risk (*N* = 92)	*P* value	Overall (*N* = 261)
Age (years)				
Mean (SD)	8.11 (5.40)	7.63 (5.02)	0.474	7.94 (5.26)
Median [min, max]	6.88 [0.918, 30.0]	6.50 [0.0329, 16.2]		6.61 [0.0329, 30.0]
Gender				
Female	85 (50.3%)	43 (46.7%)	0.675	128 (49.0%)
Male	84 (49.7%)	49 (53.3%)		133 (51.0%)
White blood cell count				
<50	169 (100%)	0 (0%)	<0.001	169 (64.8%)
≥50	0 (0%)	92 (100%)		92 (35.2%)
BCR-ABL1 fusion				
Yes	0 (0%)	5 (5.4%)	0.00967	5 (1.9%)
No	169 (100%)	87 (94.6%)		256 (98.1%)
ETV6-RUNX1 fusion				
Yes	19 (11.2%)	2 (2.2%)	0.0195	21 (8.0%)
No	150 (88.8%)	90 (97.8%)		240 (92.0%)
TCF3_HLF				
Yes	15 (8.9%)	7 (7.6%)	0.905	22 (8.4%)
No	154 (91.1%)	85 (92.4%)		239 (91.6%)
Trisomy of both chromosomes 4 and 10				
Yes	14 (8.3%)	7 (7.6%)	1	21 (8.0%)
No	155 (91.7%)	85 (92.4%)		240 (92.0%)
TCF3PBX1				
Yes	15 (8.9%)	7 (7.6%)	0.905	22 (8.4%)
No	154 (91.1%)	85 (92.4%)	239 (91.6%)	
Overall survival time (year)				
Mean (SD)	4.70 (3.26)	4.94 (3.70)	0.6	4.78 (3.41)
Median [min, max]	3.33 [0.271, 14.5]	3.21 [0.0685, 12.0]		3.33 [0.0685, 14.5]
Missing	1 (0.6%)	3 (3.3%)		4 (1.5%)
Event free survival time (years)				
Mean(SD)	2.80 (2.19)	4.05 (3.76)	0.00475	3.24 (2.89)
Median [min, max]	2.38 [0.211, 11.2]	2.54 [0.0685, 12.0]		2.39 [0.0685, 12.0]
Missing	4 (2.4%)	3 (3.3%)		7 (2.7%)

**Table 2:** The top 20 enriched KEGG pathways of RNAs involved in the ceRNA sponge regulatory network of high-risk pediatric ALL.

ID	Description	Gene ratio	*P*.adjust	*Q* value	Count
hsa05220	Chronic myeloid leukemia	16/69	1.83*E* − 16	5.80*E* − 17	16
hsa05166	Human T-cell leukemia virus 1 infection	22/69	2.96*E* − 16	9.35*E* − 17	22
hsa05161	Hepatitis B	19/69	2.44*E* − 15	7.71*E* − 16	19
hsa05206	MicroRNAs in cancer	23/69	1.43*E* − 14	4.52*E* − 15	23
hsa01522	Endocrine resistance	15/69	7.98*E* − 14	2.52*E* − 14	15
hsa05417	Lipid and atherosclerosis	19/69	2.22*E* − 13	7.03*E* − 14	19
hsa05210	Colorectal cancer	14/69	2.22*E* − 13	7.03*E* − 14	14
hsa05165	Human papillomavirus infection	22/69	3.73*E* − 13	1.18*E* − 13	22
hsa05167	Kaposi sarcoma-associated herpesvirus infection	18/69	4.16*E* − 13	1.32*E* − 13	18
hsa05212	Pancreatic cancer	13/69	7.98*E* − 13	2.52*E* − 13	13
hsa04933	AGE-RAGE signaling pathway in diabetic complications	14/69	1.26*E* − 12	3.98*E* − 13	14
hsa04660	T-cell receptor signaling pathway	14/69	2.02*E* − 12	6.40*E* − 13	14
hsa04068	FoxO signaling pathway	15/69	2.60*E* − 12	8.24*E* − 13	15
hsa05163	Human cytomegalovirus infection	18/69	3.58*E* − 12	1.13*E* − 12	18
hsa04210	Apoptosis	15/69	3.96*E* − 12	1.25*E* − 12	15
hsa05215	Prostate cancer	13/69	1.33*E* − 11	4.20*E* − 12	13
hsa04218	Cellular senescence	15/69	2.68*E* − 11	8.47*E* − 12	15
hsa05160	Hepatitis C	15/69	2.78*E* − 11	8.79*E* − 12	15
hsa05169	Epstein-Barr virus infection	16/69	8.09*E* − 11	2.56*E* − 11	16
hsa05224	Breast cancer	14/69	1.54*E* − 10	4.88*E* − 11	14

## Data Availability

The analyzed data sets generated during the study are available from the corresponding author on reasonable request.
